# Giving social support to outside family may be a desirable buffer against depressive symptoms in community-dwelling older adults: Japan gerontological evaluation study

**DOI:** 10.1186/s13030-016-0064-6

**Published:** 2016-05-24

**Authors:** Hirohito Tsuboi, Hiroshi Hirai, Katsunori Kondo

**Affiliations:** Institute of Medical, Pharmaceutical & Health Sciences, Kanazawa University, Kakuma-machi, Kanazawa, 920-1192 Japan; Faculty of Engineering, Department of Civil and Environmental Engineering, Iwate University, Iwate, Japan; Center for Preventive Medical Science, Chiba University, Chiba, Japan; Center for Well-being and Society, Nihon Fukushi University, Aichi, Japan

**Keywords:** Depression, Giving social support, Receiving social support

## Abstract

**Background:**

Depression is the leading cause of impaired quality of life and burden upon societies. Social supports can buffer against depressive symptoms effectively. The aim of this study is to determine the type of social support to have a positive relationship with depressive symptoms in healthy population.

**Methods:**

11,869 male and 12,763 female residents within the age range of 65–100 were analyzed cross-sectionally with regard to depressive symptoms (evaluated by the Japanese version of the 15-item Geriatric Depression Scale), social supports (four dimensions: giving or receiving, emotional or instrumental), and covariates utilizing data collected by the Japan Gerontological Evaluation Study. Analyzed　participants were GDS scores ≤ 10 and independence in ADL, who could give and receive supports well. Multiple linear models were applied for the analysis.

**Results:**

All supports between husband and wife were significantly associated with lower depressive degrees. In comparison with the differences between receiving and giving supports in predictive effects on depressive degrees, giving social supports to outside family, emotional or instrumental, were associated with fewer depressive symptoms.

**Conclusions:**

There is a possibility that not only supports between husband and wife but giving social supports to outside family accounts for psychological benefits against depression, in addition to supports between husband and wife.

## Background

Depression is the leading cause of disability as measured by Years Lived with Disability (YLDs) and Disability Adjusted Life Years (DALYs); especially in developed or more developed countries [1]. Depressive disorders were also identified as a leading cause of burden in the Global Burden of Disease [2]. In addition, depressive symptoms along with clinical depression are often comorbid with physical illnesses or problems such as cardiovascular disease [3], small vessel ischemia in the brain [4], inflammation [5], etc. Clinical depression may be less pervasive among the elderly [6], yet many studies have demonstrated that the prevalence of depressive symptoms increases with age [7]. Older people may express their depressive feeling by speaking of physical ailments and show more physical signs, such as weight loss, insomnia and fatigue, though they appear to be less likely to complain of affective symptoms [8]. Depression or the occurrence of depressive symptomatology is a prominent condition amongst older people, exercising a significant impact on well-being and quality of life [7].

Social supports generally possess buffering effects against depression [9]. However, it is not clear what kind of social support is beneficial; namely, under what condition the support should be offered, and which kind of interpersonal support is effective. There are somewhat inconsistent results. For example, receiving support is harmful in some instances, indicating that, for unemployed workers, the receipt of supportive messages increased suicidal ideation [10]. Another study on older subjects stated that giving emotional support to children was not associated with depressive symptoms [11]. Since the stress-buffering effects of social supports against depression depend on the stress condition of individuals [9], age, gender, marital status, and the type of social support (emotional and instrumental support; receiving and giving support) may be influential [12, 13].

We have been attempting to find a way to maintain well-being in a good cost-effective manner. If the given or received social support matches the support that the individual desires [9], social supports against depression can work effectively. The aims of this study are (1) to assess which interpersonal support is effective is connected with fewer depressive symptoms, (2) to investigate which pattern of social support (receiving or giving, instrumental or emotional) is connected with them among community-dwelling elderly people (≥65 yrs.).

## Methods

### Study population

Current analyses were based on the “Japan Gerontological Evaluation Study” (JAGES) project of 2006. The JAGES, formerly called Aichi Gerontological Evaluation Study, project aims to investigate factors related to the loss of healthy years among non-institutionalized elderly subjects aged 65 years or older, utilizing self-administrated questionnaires mailed to eligible individuals as in detailed described elsewhere [14].

Participants were all or randomly sampled residents of each municipality, who were not certificated as Needing Long-Term Care of Japan. This questionnaire was mailed to residents between March 2006 and March 2007, and 39,765 individuals (16,950 men, 20,605 women, 2,210 unknown) returned the questionnaire. The enrollment rate was 60.8 %, which is favorable compared to other studies. For data cleansing, 2,210 participants of unknown gender were excluded and 281 men and 434 women outside the age range of 65–100 were also ruled out. Consequently, 36,840 individuals (16,669 men and 20,171 women) remained. All respondents were literate and understood the Japanese language well, and were requested not to use proxy respondents. The study protocol and informed consent procedure were approved by the Ethics Committee in Research of Human Subjects at Nihon Fukushi University.

### Measures

#### Depressive symptoms

Depressive symptoms were measured using the Japanese version of the Geriatric Depression Scale (GDS), 15-point edition [15, 16].

#### Social supports

Social supports were assessed from four dimensions according to the 2-Way Social Support Scale [17, 18]. The dimensions consist of (a) receiving emotional support (RES), (b) giving (providing) emotional support (GES), (c) receiving instrumental (tangible) support (RIS), and (d) giving instrumental support (GIS). Each support was measured by a single item: “If you or others needed extra help in daily life, whom could you count on to help or to be helped by? Respondents were instructed to select appropriate answers from the following: “spouse,” “children,” and “neighbors or friends.” (a) RES was defined as a person who hears a respondent’s complaints or worries, (b) GES as a person who shares his/her complaints or worries with the respondent, (c) RIS as a person who would nurse or take care of the respondent were the respondent ill in bed for several days, and (d) GIS as a person whom the respondent would nurse or take care of were he/she ill in bed for several days. The percentage of individuals who answered each item was considered when ascertaining levels of social support.

#### Covariates

Basic activities of daily living (ADL) were evaluated by questioning “Do you perform daily activities such as walking, bathing, toileting, dressing, eating independently?” The answers were “Yes, independently”, “Need some help”, or “Always need help”. Health status variables were also evaluated in terms of comorbidity of serious diseases such as cancer, cardiovascular disease, or apoplexy. Socioeconomic status (SES) was measured in terms of years of schooling (<6, 6–9, 10–12, ≥13) and annual income. Income was defined as pre-tax annual household income including regular salary, pensions, social security, and any form of temporary earnings during a year. Income was equivalized adjusting for family size by dividing total household income by the square root of the number of people in the household [19]. For the analysis, equivalized income in Japanese Yen was divided into three categories; low (less than 1 million), middle (1–4 million), and high (4 million and over). Since income had much missing data, we created a ‘missing’ category for further analyses. The effect of living alone was included additionally as a dichotomous covariate indicating whether the respondent lives alone or lives with others at the time of survey.

### Analyzed subjects and statistical analysis

Prior to analysis, we excluded participants who may mislead the associations between depressive degrees and social supports in healthy populations. Since psychosocial factors are relatively unimportant in severe depressions [20], participants with GDS scores ≤ 10 were analyzed to the exclusion of GDS scores ≥ 11 ones. Incidentally, the Japanese version of GDS ≥ 11 represents severe depression [16]. In relation to ADL, participants who were independent in ADL were analyzed because ADL disability affects supporting behaviors, especially GIS.

The Japanese version of the IBM SPSS Statistics 19 was used for analyzing data. The data were analyzed utilizing multiple liner regression models for the adjustment of covariates that might affect results, after stratifying the data by gender and by marital status. For the comparison of depressive degrees between the married and others, analysis of covariance was utilized. For investigating variables to predict depressive symptoms, several multiple linear regression models were applied, where the score of GDS was put as a dependent variable and inserted independent variables were the type of social supports, age, comorbidity of serious diseases, living status, equivalent income, and years of schooling. Significance is reported at *p* value < .05. Standardized beta coefficients (*B*) are also presented.

## Results

We summarize the demographic data of all participants and the analyzed subjects in Table 1 for displaying characteristics of sampled and analyzed data.

**Table 1:** Characteristics of participants

	All participants		Analyzed participants	
	Men (*n*=16,669)	Women (*n*=20,171)	Men (*n*=11,869)	Women (*n*=12,763)
Age (mean (SD), years)	72.9 (5.92)	73.5 (6.33)	72.8 (5.89)	73.5 (6.32)
GDS scores (Mean (SD))	3.45 (3.340)	3.61 (3.359)	2.91 (2.638)	3.07 (2.656)
GDS scores	n (%)	n (%)	n (%)	n (%)
0	2,375 (14.2)	2,165 (10.7)	2,196 (18.5)	1,966 (15.4)
1	2,587 (15.5)	2,747 (13.6)	2,346 (19.8)	2,514 (19.7)
2	2,143 (12.9)	2,386 (11.8)	1,953 (16.5)	2,150 (16.8)
3	1,556 (9.3)	1,804 (8.9)	1,400 (11.8)	1,617 (12.7)
4	1,204 (7.2)	1,351 (6.7)	1,079 (9.1)	1,232 (9.7)
5	892 (5.4)	1,011 (5.0)	784 (6.6)	910 (7.1)
6	718 (4.3)	753 (3.7)	654 (5.5)	675 (5.3)
7	565 (3.4)	616 (3.1)	501 (4.2)	547 (4.3)
8	439 (2.6)	505 (2.5)	386 (3.3)	454 (3.6)
9	366 (2.2)	434 (2.2)	315 (2.7)	388 (3.0)
10	283 (1.7)	340 (1.7)	255 (2.1)	310 (2.4)
11	242 (1.5)	291 (1.4)		
12	193 (1.2)	233 (1.2)		
13	142 (0.9)	171 (0.8)		
14	105 (0.6)	123 (0.6)		
15	50 (0.3)	36 (0.2)		
Unknown	2,809 (16.9)	5,205 (25.8)		
Medical treatment	n (%)	n (%)	n (%)	n (%)
Cancer, cardiovascular disease or apoplexy	3,561 (21.4)	2,967 (14.7)	2,408 (20.3)	1,772 (13.9)
None or unknown	13,108 (78.6)	17,204 (85.3)	9,461 (79.7)	10,991 (86.1)
Basic activities of daily living	n (%)	n (%)	n (%)	n (%)
Independent	15,560 (93.3)	18,938 (93.9)	11,869 (100)	12,763 (100)
Needs help	394 (2.4)	732 (3.6)		
Unknown	715 (4.3)	501 (2.5)		
Marital status	n (%)	n (%)	n (%)	n (%)
Married^a^	14151 (84.9)	10878 (53.9)	10673 (89.9)	7552 (59.2)
Widowed (<1 year)	263 (1.6)	855 (4.2)	179 (1.5)	477 (3.7)
Widowed (≥1 year)	1133 (6.8)	6255 (31.0)	739 (6.2)	4047 (31.7)
Divorced	251 (1.5)	539 (2.7)	175 (1.5)	350 (2.8)
Unmarried	145 (0.9	504 (2.5)	103 (0.9)	337 (2.6)
Unknown	726 (4.0)	1140 (6.0)		
Live alone	n (%)	n (%)	n (%)	n (%)
Yes	824 (4.9)	3028 (15.0)	542 (4.6)	1831 (14.3)
No	15845 (95.1)	17143 (85.0)	11327 (95.4	10932 (85.7)
Equivalent income (Japanese Yen)	n (%)	n (%)	n (%)	n (%)
<1 million	1244 (7.5)	2199 (10.9)	766 (6.5)	1,368 (10.7)
1 - 4 million	7097 (42.6)	6284 (31.2)	5,471 (46.1)	4,652 (36.4)
≥4 million	919 (5.5)	835 (4.1)	743 (6.3)	675 (5.3)
Unknown	7409 (44.4)	10853 (53.8)	4,889 (41.2)	6,068 (47.5)
Years of schooling	n (%)	n (%)	n (%)	n (%)
<6 years	328 (2.0)	838 (4.2)	166 (1.4)	434 (3.4)
6 - 9 years	8325 (49.9)	10676 (52.9)	5,701 (48.0)	6,616 (51.8)
10 - 12 years	5053 (30.3)	6508 (32.3)	3,849 (32.4)	4,440 (34.8)
≥13 years	2629 (15.8)	1545 (7.7)	2,040 (17.2)	1,091 (8.5)
Unknown	334 (2.0)	604 (3.0)	113 (1.0)	182 (1.0)
Social supports	%	%	%	%
Receiving emotional support				
from partner^a^	78.1	66.0	80.0	69.2
from children	29.2	50.4	30.4	53.5
from outside family	24.5	43.3	26.1	46.3
Giving emotional support				
to partner^a^	75.7	55.2	77.6	58.0
to children	32.6	41.9	35.0	45.6
to outside family	28.1	48.5	30.5	52.8
Receiving instrumental support				
from partner^a^	92.2	76.8	93.2	79.1
from children	40.2	63.9	41.9	67.2
from outside family	3.2	8.5	3.4	9.1
Giving instrumental support				
to partner^a^	91.7	88.4	93.3	90.4
to children	40.1	55.1	43.0	60.7
to outside family	8.0	17.1	8.5	18.9

^a^Denominators of partner supports were married participants

### Distribution of depressive symptoms by gender and marital status

**Fig. 1:**
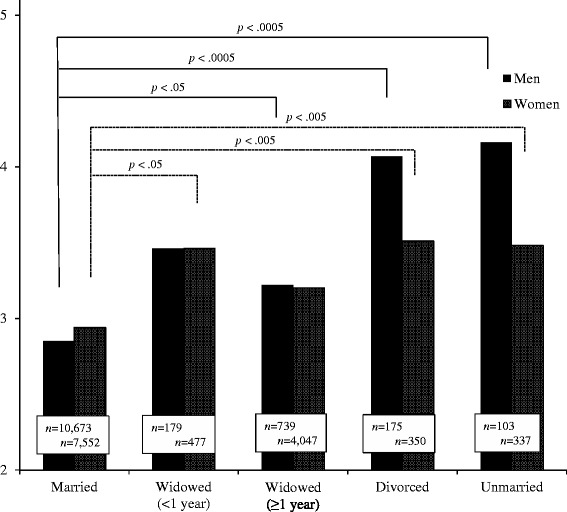
Depressive symptoms assessed by the 15-item version of the Geriatric Depression (GDS) Scale according to marital status and gender. Analyzed subjects are GDS scores ≤ 10 and independence in ADL. Comparisons of GDS scores were made between married subjects and those in other marital status by gender. All comparisons were controlled for age, socioeconomic status (equivalent income and years of schooling), living status, and comorbidity of serious diseases by linear analysis models. Solid lines indicate significant differences among male strata; dotted lines those among female strata

Figure 1 shows the degree of depressive symptoms assessed by GDS according to gender and marital status brackets, where age, SES, living status and comorbidity of serious diseases were controlled in comparing depressive levels. In the male strata, the married showed significantly lower depressive symptoms in comparison with the widowed ≥ 1 year, the divorced, and the unmarried (*B* = .019, *p* < .05; *B* = .060, *p* < .0005; *B* = .050, *p* < .0005, respectively), whereas no significant difference was seen between the married and the widowed < 1 year. In the female strata, depressive symptoms of the married were significantly lower than those of the widowed < 1 year, the divorced, and the unmarried (*B* = .028, *p* < .05; *B* = .036, *p* < .005; *B* = .036, *p* < .005, respectively), whereas no significant difference was seen between the married and the widowed ≥ 1 year.

#### Four dimensions of social supports

**Fig. 2:**
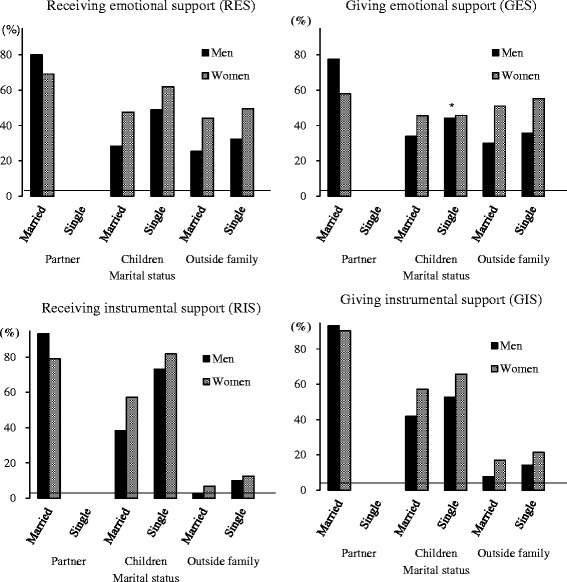
Social supports by marital status and gender

In consideration of the above-mentioned results regarding depressive symptoms, we merge the widowed, the divorced, and the unmarried into one variable; consequently, analyses were performed by two marital strata (married and others). Figure 2 represents four dimensions of social support to and from partner, children, and outside family by marital status and by gender.

#### Variables to predict the degrees of depressive symptoms

Table 2 presents univariate analyses on each covariate predicting depressive symptoms. Age was related with higher depressive symptoms, though the univariate regression model among single men did not significantly fit (*F* = .71, *p* = .40). Comorbidity of serious diseases predicted depressive symptoms among all strata. Live alone showed positive associations with depressive symptoms among men. On the contrary, higher equivalent income and longer years of schooling predicted lower depressive symptoms.

**Table 2:** Univariate regression values of each covariate predicting depressive symptoms

Marital status	Married						Single					
Gender	Men (*n*=10,673)			Women (*n*=7,552)			Men (*n*=1,196)			Women (*n*=5,211)		
	*B*	*p*	95 % CI	*B*	*p*	95 % CI	*B*	*p*	95 % CI	*B*	*p*	95 % CI
Age												
(continuous variable)	0.096	***	0.04 ; 0.05	0.080	***	0.03; 0.05	^a^			0.130	***	0.04 ; 0.06
Comorbidity of serious diseases												
Under medical treatment	0.091	***	0.47 ; 0.71	0.101	***	0.60; 0.94	0.067	*	0.08; 0.89	0.082	***	0.42 ; 0.84
Living status												
Live alone	0.034	**	0.47 ; 1.67	0.017		−0.17; 1.33	0.076	*	0.11; 0.78	0.003		−0.14 ; 0.18
Equivalent income (Japanese yen)												
<1 million	(reference)			(reference)			(reference)			(reference)		
1 - 4 million	−0.199	***	−1.25; −0.83	−0.183	***	−1.16; −0.76	−0.219	***	−1.97; −0.66	−0.159	***	−1.21 ; −0.69
≥4 million	−0.169	***	−2.09; −1.54	−0.133	***	−1.86; −1.24	−0.188	***	−3.14; −1.37	−0.107	***	−1.69 ; −0.91
Years of schooling												
<6 years	(reference)			(reference)			(reference)			(reference)		
6 - 9 years	−0.208	***	−1.52 ; −0.65	−0.167	**	−1.36 ;−0.37	−0.194	*	−2.20; −0.04	−0.146	***	−1.11 ; −0.48
10 - 12 years	−0.292	***	−2.06; −1.18	−0.220	***	−1.69 ;−0.70	−0.202	*	−2.37; −0.17	−0.186	***	−1.39 ; −0.75
≥13 years	−0.272	***	−2.30 ; −1.41	−0.168	***	−2.02; −0.97	−0.170	*	−2.60; −0.30	−0.129	***	−1.75 ; −0.94

"Single" includes widow/widower, the unmarried and the divorced

B: standardized beta, p : * < .05, ** < .005, *** .0005

^a^Regression model has no predictive capability (*p* value for *F* test ≥ .05)

Table 3 exhibits to what extent each support explained depressive symptoms under the influence of the above-mentioned covariates. With the exception of RIS from outside family among men and GIS to outside family among single men, any supports predicted significantly lower depressive symptoms.

**Table 3:** Each support predicting depressive symptoms under the influence of covariates

Marital status	Married						Single					
Gender	Men (*n*=10,673)			Women (*n*=7,552)			Men (*n*=1,196)			Women (*n*=5,211)		
	*B*	*p*	95 % CI	*B*	*p*	95% CI	*B*	*p*	95 % CI	*B*	*p*	95 % CI
Social supports												
Receiving emotional support												
from partner	-.093	***	−0.73 ; −0.49	-.122	***	−0.81 ; −0.56						
from children	-.030	**	−0.28 ; −0.07	-.023	*	−0.24 ; −0.01	-.069	*	−0.73 ; −0.07	-.091	***	−0.66 ; −0.36
from outside family	-.077	***	−0.57 ; −0.35	-.058	***	−0.42 ; −0.18	-.098	**	−0.95 ; −0.26	-.073	***	−0.55 ; −0.25
Giving emotional support												
to partner	-.090	***	−0.68 ; −0.44	-.099	***	−0.63 ; −0.40	-.168	*	−2.57 ; −0.28	-.082	***	−1.27 ; −0.45
to children	-.066	***	−0.46 ; −0.26	-.034	**	−0.29 ; −0.06	-.085	**	−0.81 ; −0.16	-.088	***	−0.63 ; −0.34
to outside family	-.098	***	−0.66 ; −0.45	-.096	***	−0.61 ; −0.38	-.133	***	−1.13 ; −0.46	-.132	***	−0.88 ; −0.57
Receiving instrumental support												
from partner	-.073	***	−0.95 ; −0.56	-.107	***	−0.83 ; −0.54						
from children	-.070	***	−0.48 ; −0.28	-.053	***	−0.39 ; −0.16	-.176	***	−1.51 ; −0.77	-.112	***	−0.98 ; −0.60
from outside family	-.010		−0.46 ; 0.15	-.050	***	−0.74 ; −0.28	-.007		−0.61 ; 0.48	-.071	***	−0.81 ; −0.36
Giving instrumental support												
to partner	-.090	***	−1.14 ; −0.75	-.093	***	−1.02 ; −0.62						
to children	-.076	***	−0.50 ; −0.30	-.078	***	−0.53 ; −0.29	-.157	***	−1.22 ; −0.58	-.149	***	−1.02 ; −0.70
to outside family	-.057	***	−0.73 ; −0.37	-.070	***	−0.64 ; −0.33	-.048		−0.87 ; 0.07	-.116	***	−0.95 ; −0.59

"Single" includes widow/widower, the unmarried and the divorced

B: standardized beta, p : * < .05, ** < .005, *** .0005

Table 4 displays the findings of a multiple regression model that assessed variables to predict depressive symptoms, where all variables in relation to social supports and covariates were included as independent variables in order to investigate meaningful interpretive output. Since all support variables were put into one model, subtle associations among each variable were presented. However, in order to avoid multicollinearity problems and to identify the directional effects of social supports, we put giving and receiving support variables of each category (emotional/instrumental, partner/children/outside family) into one model with covariates; followed by comparison between giving and receiving supports in each category (Table 5). Although four kinds of supports between husband and wife were significant predictors against depressive symptoms, GIS (*B* = −.073) appeared to be a stronger predictor than RIS (*B* = −.042) among men, whereas receiving supports appeared stronger predictors than giving supports among other strata. In relation to support involving children, GES was associated with lower depressive symptoms in comparison with RES with the exception of single men　(Table 5). With respect to social support with outside family, giving supports, emotional or instrumental, were stronger predictors against depressive symptoms in comparison with receiving supports with the exception of single male stratum (Table 5).

**Table 4:** Multiple regression model predicting depressive symptoms

Marital status	Married						Single					
Gender	Men (*n*=10,673)			Women (*n*=7,552)			Men (*n*=1,196)			Women (*n*=5,211)		
	*B*	*p*	95 % CI	*B*	*p*	95 % CI	*B*	*p*	95 % CI	*B*	*p*	95 % CI
Social supports												
Receiving emotional support												
from partner	-.041	**	−0.43 ; −0.10	-.068	***	−0.55 ; −0.21						
from children	.045	***	0.12 ; 0.41	.018		−0.06 ; 0.24	.028		−0.27 ; 0.60	-.028		−0.35 ; 0.04
from outside family	-.014		−0.24 ; 0.07	.014		−0.09 ; 0.23	-.006		−0.51 ; 0.44	.028		−0.05 ; 0.35
Giving emotional support												
to partner	-.038	**	−0.39 ; −0.09	-.033	*	−0.33 ; −0.03						
to children	-.040	**	−0.36 ; −0.08	.005		−0.13 ; 0.18	-.023		−0.57 ; 0.30	-.043	*	−0.42 ; −0.05
to outside family	-.074	***	−0.57 ; −0.27	-.093	***	−0.65 ; −0.32	-.112	**	−1.14 ; −0.21	-.119	***	−0.86 ; −0.45
Receiving instrumental support												
from partner	-.024	*	−0.47 ; −0.02	-.058	***	−0.54 ; −0.19						
from children	-.041	**	−0.36 ; −0.09	-.044	**	−0.38 ; −0.08	-.129	***	−1.29 ; −0.39	-.037	*	−0.49 ; −0.03
from outside family	.030	**	0.15 ; 0.84	-.015		−0.42 ; 0.11	.040		−0.31 ; 1.07	-.015		−0.39 ; 0.15
Giving instrumental support												
to partner	-.051	***	−0.76 ; −0.32	-.040	**	−0.57 ; −0.13						
to children	-.011		−0.19 ; 0.07	-.031	*	−0.31 ; −0.01	-.077	*	−0.83 ; −0.06	-.096	***	−0.74 ; −0.37
to outside family	-.033	**	−0.53 ; −0.10	-.020	.141	−0.33 ; 0.05	-.030		−0.85 ; 0.36	-.075	***	−0.73 ; −0.27

*B*: standardized beta, *p* : * < .05, ** < .005, *** .0005

Model was controlling for age, comorbidity of serious diseases, living status, equivalent income, years of schooling

**Table 5:** Multiple regression models for comparing the effects of receiving and giving supports to predict depressive symptoms

Marital status	Married						Single					
Gender	Men (*n*=10,673)			Women (*n*=7,552)			Men (*n*=1,196)			Women (*n*=5,211)		
	*B*	*p*	95 % CI	*B*	*p*	95 % CI	*B*	*p*	95 % CI	*B*	*p*	95 % CI
Emotional supports												
Model 1-1												
Partner												
Receiving (RES)	-.061	***	−0.55 ; −0.24	-.099	***	−0.71 ; −0.40						
Giving (GES)	-.051	***	−0.47; −0.17	-.039	*	−0.35 ; −0.06						
Model 1-2												
Children												
Receiving (RES)	.020		−0.03 ; 0.25	-.004		−0.17 ; 0.13	-.024		−0.57 ; 0.29	-.060	***	−0.52 ; −0.16
Giving (GES)	-.078	***	−0.56 ; −0.30	-.031	*	−0.31 ; −0.01	-.070		−0.83 ; 0.02	-.055	**	−0.48 ; −0.12
Model 1-3												
Outside family												
Receiving (RES)	-.017		−0.26 ; 0.06	.013	*	−0.09 ; 0.23	-.015		−0.56 ; 0.37	.018		−0.10 ; 0.29
Giving (GES)	-.086	***	−0.64 ; −0.34	-.105	***	−0.70 ; −0.38	-.123	**	−1.19 ; −0.28	-.143	***	−0.98 ; −0.59
Instrumental supports												
Model 2-1												
Partner												
Receiving (RIS)	-.042	***	−0.65 ; −0.22	-.084	***	−0.69 ; −0.38						
Giving (GIS)	-.073	***	−0.97 ; −0.54	-.061	***	−0.75 ; −0.32						
Model 2-2												
Children												
Receiving (RIS)	-.039	**	−0.33 ; −0.08	-.016		−0.22 ; 0.05	-.128	***	−1.26 ; −0.41	-.053	**	−0.59 ; −0.16
Giving (GIS)	-.052	***	−0.40 ; −0.15	-.069	***	−0.50 ; −0.22	-.095	**	−0.91 ; −0.18	-.123	***	−0.89 ; −0.53
Model 2-3												
Outside family												
Receiving (RIS)	.024	*	0.04 ; 0.73	-.019		−0.46 ; 0.07	.038		−0.33 ; 1.06	-.009		−0.35 ; 0.20
Giving (GIS)	-.068	***	−0.87 ; −0.46	-.061	***	−0.60 ; −0.24	-.072		−1.19 ; 0.00	-.110	***	−0.96 ; −0.51

*B*: standardized beta, *p* : * < .05, ** < .005, *** .0005

Model was controlling for age, comorbidity of serious diseases, living status, equivalent income, years of schooling

Receiving and giving supports in every category along with covariates were added into each regression model

## Discussion

We assessed depressive symptoms and social supports of older residents in the central area of Japan, as well as confounding factors such as age, equivalent income, years of education, whether to live alone, and comorbidity of serious diseases according to living status and gender cross-sectionally, utilizing a substantial amount of data. Although almost all kinds of social supports predict fewer depressive symptoms (Table 3), we compared directions of the social supports (receiving or giving) for exploring more valid factor against depression. We found that social supports giving outside family were stronger predictor against depressive symptoms in comparison with receiving them (Table 5). In the relation to husband and wife, spousal supports were significant factors against depressive symptoms, though there were some, if any, differences between receiving and giving supports against depressive symptoms. In addition, SES (equivalent income and years of schooling) was a predictive factor for fewer depressive symptoms among all strata.

### Depressive symptoms and SES

As shown in Table 2, SES is a predictive factor for depressive symptoms. This result appears to contradict a report prepared by the Ministry of Health and Welfare of Japan [21], which mentions that the potential relationships between depressive symptoms and SES in Japan are not clear. It used to be the case that there were no studies on large subjects. However, there are recent studies that have reported the relationships between SES and depressive symptoms among the Japanese population. For instance, JAGES data (almost the same cohort as this study) of 2003 [22] and J-HOPE (the Japanese study of Health, Occupation and Psychosocial factors related Equity) study discovered relationships between depressive state and SES [23]. With regard to income, subjective sense of low economic status was reportedly associated with depressive state or psychosocial deterioration in community-living elderly people [24]. These studies support our present results.

#### Supports between husband and wife to predict depressive symptoms

In relation to social supports among the married, all supports to and from the partner were related to lower depressive symptoms in all strata (Table 5). It is understandable to be relieved and less depressive when one’s partner is responsive. However, the results were somewhat different from what we had expected: it is a common belief of older people that a husband does not hear his wife and that a wife devotes herself to her husband in daily life. That is, men generally receive substantially more instrumental advantages from marriage than women do in the form of housekeeping services [25, 26]. Men may also receive more emotional advantages from marriage. Despite of these expectations, GIS and RIS to one’s wife among married men were significantly related to lower depressive symptoms as shown in Tables 3, 4 and 5. These findings may indicate an inner voice whereby a husband thinks that he should hear his wife because he often insists on attention or issues orders toward her, while a wife may be glad of her husband’s instrumental support because her husband rarely helps her, and that wives may be tired of listening to their husbands. Taken together, supports with partner are good predictor against depressive symptoms (Tables 3 and 5).

#### Social supports with adult children to predict depressive symptoms

We analyzed supports involving children living together and living separately as a whole, because there were no differences in the analysis between the two groups (data not shown). Although all kinds of supports to and from children predict lower depressive symptoms as shown in Table 3, influence degree of RES from children on depressive symptoms was lower in comparison with other supports (Tables 3 and 5). This result may indicate the difficulty of intergenerational relations [27] or an ambivalent perspective toward children that causes conflicts [28]. It may be that a negative part of RES (talking to children) includes parents’ complaints to children. Although one study performed on 52 widowers and 44 widows found that both types of social support from adult children might have negative consequences under certain conditions, the supports were instrumental and emotional ones from children [29]. Without specifying the provision of social support, studies suggest that intergenerational social support appears to be minimally positive and may even have negative effects on the psychological well-being of older parents [11, 29]. Intergenerational support between parents and adult children can therefore be complicated,though the current study indicated desirable results of supports to and from adult children as a whole (Table 3).

#### Social supports with outside family to predict depressive symptoms

It is interesting that giving supports to outside family were stronger relationships with depressive symptoms in comparison with receiving support from outside family as shown in Table 5, though both supports to and from outside family themselves predict lower depressive symptoms (Table 3). We are not aware of any other studies describing the same results. However, there are similar studies confirming that giving something to others is beneficial to an individual. Cross-sectional and longitudinal studies have shown that spending income on others is more strongly associated with happiness in comparison with personal spending [30]. Happiness, or positive feelings, can work as a buffer against depression. It was indicated that happiness intervention was associated with decreased depression [31], and the pursuit of happiness leads one to infrequently experiencing negative emotions [32]. Given that giving something to others stir sympathy or it is conceivable as altruistic behavior, there are some reports of great interest. For example, a biopsychological study utilizing a virtual ball-toss task revealed that sympathetic concern behavior toward others arouses positive feelings [33]. Altruistic attitudes or helping behaviors proved to be significant predictors of positive effects on mental health concerns such as depressive symptoms and negative mental health among elderly individuals in general [34], though altruistic behaviors pose a harmful effect on major depressive patients [35]. In addition, it may sound as a favorable interpretation, the following two reports can explain our present results. A longitudinal epidemiological study on older adults indicated that provision of social support to others reduced the risk of mortality, whether the support was operationalized as instrumental support provided to neighbors, friends, or relatives [10]. Since aging interacts with depression to enhance risks for morbidity and mortality [36], giving support may also become a protective factor against depression. Giving supports, which can constitute one form of altruistic behavior, appears to have a positive effect on non-clinical depressive symptoms. The reason why the positive associations between giving supports and depressive symptoms was indicated only among outside family can be that there was no negative bondage or constraints such as intergenerational problems or complaints among close relationships. It is possible that we can choose our friends but not our family or relatives. The interpersonal quality of social contacts may thus be effective on mental health.

## Limitations

This study has both methodological strengths and limitations. Its strengths include the following: it was based on a large population and several important factors that can influence depressive symptoms were assessed and included in a multivariate analysis. Limitations were as follows. Firstly, this study relied on self-reported measures of all variables. Some concern about recall and report bias, in which depressed subjects may remember and report more negatively about their mental conditions and social supports on a systematic basis, is warranted. Secondly, this study is based on cross-sectional data, which may preclude definite conclusions regarding causal relationships among variables. Longitudinal data are necessary to further unravel the complex interplay between the course of depressive symptoms, widowhood, and social supports. Nevertheless, the results of the present study suggest the social support patterns that can buffer depressive symptoms among older men and women.

## Conclusions

We analyzed factors that can have desirable associations with depressive state in older adults. Although age, having serious diseases, SES, living alone (only among men) and social　supports were important factors to predict higher or lower depressive symptoms, the former three variables are difficult to change. By contrast, social supports are ameliorable factors. Therefore, we explored patterns of social supports in detail.

Our findings paint a picture: first, compared with men, women are more likely to have supports outside of the conjugal relationship; second, men can be more dependent on their wives in terms of their psychological wellbeing, and this may lead them to become more depressive after the bereavement. Furthermore, we found an important factor: giving support to outside family can be a better buffer against depression than receiving support in both men and women. Future research, such as a longitudinal study, would do best to focus on pathways of social supports against depression.
